# Supplementary material for: Crosstalk between Existential Phenomenological Psychotherapy and Neurological Sciences in Mood and Anxiety Disorders
Source: Biomedicines. 2021 Mar 27;9(4):340. doi: 10.3390/biomedicines9040340 (PMC8066576; doi:10.3390/biomedicines9040340)
Supplement: Supplementary file 1 [file biomedicines-09-00340-s001.pdf]

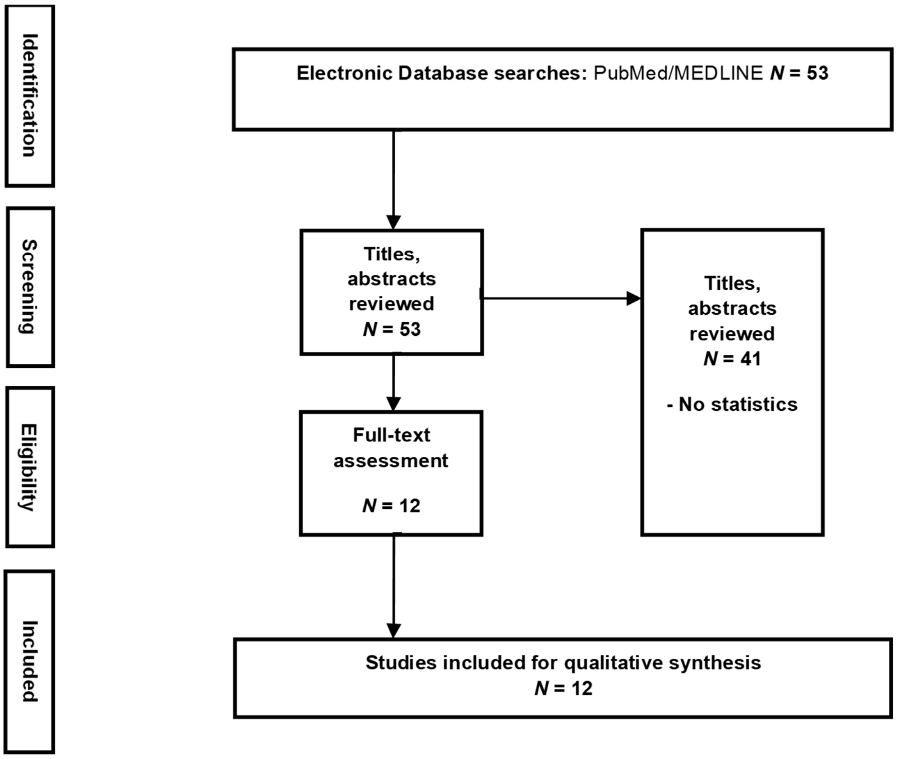

Figure S1. Preferred reporting items for systematic reviews and meta-analysis (PRISMA) flow diagram for systematic review
